# Antifungal activity of well-defined chito-oligosaccharide preparations against medically relevant yeasts

**DOI:** 10.1371/journal.pone.0210208

**Published:** 2019-01-08

**Authors:** Monica Ganan, Silje B. Lorentzen, Jane W. Agger, Catherine A. Heyward, Oddmund Bakke, Svein H. Knutsen, Berit B. Aam, Vincent G. H. Eijsink, Peter Gaustad, Morten Sørlie

**Affiliations:** 1 Department of Chemistry, Biotechnology, and Food Science, Norwegian University of Life Sciences, Aas, Norway; 2 Institute of Clinical Medicine, Department of Microbiology, University of Oslo, Blindern, Oslo, Norway; 3 Department of Biosciences, University of Oslo, Blindern, Oslo, Norway; 4 Nofima, Norwegian Institute of Food Fisheries & Aquaculture Research, Aas, Norway; University of South Carolina, UNITED STATES

## Abstract

Due to their antifungal activity, chitosan and its derivatives have potential to be used for treating yeast infections in humans. However, to be considered for use in human medicine, it is necessary to control and know the chemical composition of the compound, which is not always the case for polymeric chitosans. Here, we analyze the antifungal activity of a soluble and well-defined chito-oligosaccharide (CHOS) with an average polymerization degree (DP_n_) of 32 and fraction of acetylation (F_A_) of 0.15 (C32) on 52 medically relevant yeast strains. Minimal inhibitory concentrations (MIC) varied widely among yeast species, strains and isolates (from > 5000 to < 9.77 μg mL^-1^) and inhibition patterns showed a time- and dose-dependencies. The antifungal activity was predominantly fungicidal and was inversely proportional to the pH, being maximal at pH 4.5, the lowest tested pH. Furthermore, antifungal effects of CHOS fractions with varying average molecular weight indicated that those fractions with an intermediate degree of polymerization, i.e. DP 31 and 54, had the strongest inhibitory effects. Confocal imaging showed that C32 adsorbs to the cell surface, with subsequent cell disruption and accumulation of C32 in the cytoplasm. Thus, C32 has potential to be used as a therapy for fungal infections.

## Introduction

The frequency of yeast infections in humans has increased during the last decades mostly due to the growing number of immunocompromised patients and to the emergence of antifungal resistance [1]. *Candida albicans* is often considered the predominant cause of invasive and superficial fungal infections, but the epidemiology of yeast infections is rapidly evolving and other yeasts have emerged as major opportunistic pathogens [2, 3]. The spread of multidrug-resistant yeast strains and the reduced number of effective drugs available make it necessary to find new antifungal substances.

Chitosan is a cationic linear heteropolymer produced by partial deacetylation of chitin, the most abundant natural polysaccharide in Nature after cellulose [4]. Chitosan is composed of a variable number of *β*-(1–4) linked units of 2-acetamide-2-deoxy-*β*-d-glucopyranose (GlcNAc) and 2-amino-2-deoxy-*β*-d-glycopyranose (GlcN) [5]. The number of monomeric units defines the degree of polymerization (DP) of a chitosan, while the fraction of acetylation (*F*_A_) is a measure of the average number of GlcNAc relative to the sum of GlcN and GlcNAc. Both, *F*_A_ and DP are fundamental for some key physical-chemical properties, including solubility and conformation [6–8]. Importantly, polymeric chitosan may be chemically or enzymatically converted to shorter fragments, or chito-oligosaccharides (CHOS), with markedly different physicochemical properties such as higher solubility and lower viscosity, and have several appealing biological activities [9, 10]. Obtaining well-defined CHOS preparations, especially preparations of longer CHOS (DP_n_ > 10) is not straightforward.

Chitosan and CHOS are considered to be biodegradable, non-toxic, non-immunogenic and non-carcinogenic. Also, they are biologically compatible and chemically versatile [5]. These properties make chitosan well-suited for a wide range of biomedical applications such as in drug delivery, wound healing, neural stem cell growth, tissue engineering, gene therapy, and treatment of infections [11]. Moreover, CHOS and chitosan have antibacterial and antifungal activity, affecting growth of a wide range of bacteria, yeasts and molds, mainly affecting agriculture and the food industry [12, 13]. Only few reports have discussed the effects of chitosan and CHOS on *C*. *albicans* and other medically relevant yeasts [14–18], and the conclusions of existing studies are sometimes inconsistent. Also, in general, these previous studies were not based on chemically defined CHOS, which hampers the establishment of structure-function relationships and hinders the potential approval of CHOS as a therapy for medical purposes [13, 19, 20].

The main aim of the present study was to evaluate the inhibitory effects of a well-defined CHOS mixture on yeasts strains commonly involved in human infections, and to better understand the inhibition mechanism. With this purpose, we have carried out an in-depth analysis of the effects a well-defined CHOS preparation (C32; DP_n_ = 32, *F*_A_ = 0.15) with good water solubility and known antifungal activity on medically relevant yeasts [19] as well as some additional trials with some selected oligosaccharide preparation thereof.

## Materials and methods

### Enzymatic production of CHOS

CHOS with DP_n_ of 32 and *F*_A_ of 0.15 as determined by ^1^H-NMR (abbreviated C32) were prepared as described previously [13]. For subfractionation, C32 was dissolved in water to a concentration of 20 mg/mL and dialyzed against distilled water using Spectra/Por 6 dialysis membranes with cutoffs of 3.5 kDa, 8.0 kDa, 10 kDa, and 15 kDa (Spectrumlabs, Rancho Dominguez, CA, USA). Each dialysis step was performed at 4°C against water with stirring for 48h. At the end of each dialysis step, the retentate and/or permeate was collected and lyophilized. Prior to use in biological experiments, the CHOS were dissolved in two-fold concentrated culture medium and sterilized by filtration.

### Determination of average degree of polymerization (DP_n_) with ^1^H-NMR spectroscopy

^1^H NMR experiments were performed on an Avance^TM^ 400 instrument from Bruker. The DP_n_ was calculated by the equation (Dα+Dβ+D+Aα+Aβ+A)/(Dα+Dβ+Aα+Aβ), where Dα, Dβ, Aα and Aβ are the integral of the reducing end signals of the α and β anomers of the deacetylated (GlcN, D) and acetylated (GlcNAc, A) units, D is the integral of the signals from the internal and nonreducing end deacetylated units and A is the integral of the signals from the internal and non-reducing end acetylated units. For experimental details and assignments of the signals used for quantification please refer to Sørbotten *et al*. 2005 [20].

### Determination of relative molecular weights of CHOS fractions

Size exclusion chromatography was performed on a Dionex Ultimate 3000RSLC system (ThermoScientific, Sunnivale USA) with RI detection. The columns were a TOSOH TSKgel G3000PWXL-CP (7.8 x 300 mm, 7 μm) and a TOSOH TSKgel G-oligoPW (7.8 x3 00 mm, 7 μm) coupled in series and where operated isocratically at 1 mL/min with 0.1 M NaNO_3_ as the mobile phase. Samples were dissolved in the mobile phase. The system was calibrated with pullulan standards with molecular masses of 6 kDa, 12 kDa, 22 kDa, 50 kDa and 110 kDa (PSS Polymer Standards Service, Mainz, Germany). Chromatography data were exported and processed by WinGPC Scientific v 6.20 software for calculation of average molecular weight (MW) using linear calibration.

### 2-Aminoacridone derivatization of chito-oligosaccharides

The reductive amination of chito-oligosaccharides with 2-aminoacridone (AMAC) was performed as previously described [21, 22].

### Yeast strains

*In vitro* growth inhibition tests were performed on 42 clinical isolates belonging to the Oslo University Hospital Collection and comprising the following species: *Candida albicans* (n = 3), *Candida glabrata* (n = 3), *Candida guilliermondii* (n = 3), *Candida inconspicuae* (n = 1), *Candida kefyr* (n = 3), *Candida krusei* (n = 3*)*, *Candida lusitaniae* (n = 3), *Candida neoformans* (n = 4), *Candida norvegensis* (n = 3), *Candida parapsilosis* (n = 3), *Candida pelliculosa* (n = 1), *Candida sorbosa* (n = 1), *Candida tropicalis* (n = 3), *Rhodotorula glutinis* (n = 1), *Rhodotorula mucilaginosa* (n = 3), *Schizosaccharomyces pombe* (n = 1), and *Saccharomyces serevisiae* (n = 3). Here, n refers to the number of independent clinical isolates of individual fungal species. In addition, *C*. *albicans* 90028 and 10231, *C*. *glabrata* 15545, *C*. *guilliermondii* 6260, *C*. *inconspicuae* 96273, *C*. *krusei* 6258, *C*. *lusitaniae* 34449, *C*. *norvegensis* 22977, *C*. *parapsilosis* 22019, and *C*. *tropicalis* 13803 from the American Type Culture Collection (ATCC) were used. The strains, 52 in total, were kept frozen in YPD broth and glycerol at -70°C until testing. For each experiment, yeasts were subcultured on Sabouraud agar and incubated for 48 h at 37°C.

### Preparation of inocula

Yeast cell suspensions were prepared in sterile water by touching ten colonies from a culture plate and adjusting the resulting suspension to 0.5 McFarland turbidity standard (approximately 7 x 10^6^ CFU mL^-1^) using spectrophotometric methods. One milliliter of the cell suspension was then added to 9 mL of two-fold concentrated RPMI-1640, providing the starting inoculum of approximately 7 x 10^5^ CFU mL^-1^.

### Growth inhibition assays

A microdilution broth method was used to determine the minimal inhibitory concentration [1] values for yeasts [23]. Briefly, 100 μL of yeast inoculum obtained as previously described were added to a 96-well microplate containing different combinations of C32 in potato dextrose agar (PDA) to a total volume of 200 μL, yielding final concentrations 5000 μg.mL^-1^ to 9.8 μg.mL^-1^. The minimal inhibitory concentration (MIC) was defined as the lowest drug concentration at which there was no visible growth after 48 h incubation at 37°C. To obtain the minimal fungicidal concentration (MFC) after 48 h incubation, 10 μL of each serial dilution were taken from every well in which no visible growth was observed and spread on Sabouraud agar. Plates were incubated at 37°C for 48 h. The dilution of the samples was such that the detection limit of the method was 1 x 10^3^ CFU mL^-1^. The MFC was defined as the lowest drug concentration that yielded ten or fewer colonies. C32 was considered fungicidal if the MFC to MIC ratio was ≤ 4, and fungistatic if the ratio was > 4 [24].

### Time-kill curve procedures

Yeast inocula obtained as previously described were incubated using the microdilution broth method in the absence (control) or presence of different C32 concentrations (0.25 x, 0.5 x, 1 x, 2 x, 5 x, and 10 x MIC) for a period of 24 h at 37°C. Samples were taken at regular intervals to record survival. Colony counts were determined after incubation on Sabouraud agar at 37°C for 24 to 48 h. Log CFU mL^-1^ values were plotted against time for each concentration of C32 tested.

### Effect of temperature

The influence of temperature, 37°C (physiological temperature) and 41.5°C (fever temperature) on the antimicrobial activity of chitosan was determined according to the previously mentioned method for MIC determination.

### Yeast labeling

Yeast cell suspensions were prepared as previously described and incubated in RPMI for 24 h at 37°C with different concentrations of AMAC-C32. Non-labeled C32 and water were used as controls. Ethanol [50% (v/v) final concentration] was used as cell wall disruption control. After this 24 h incubation, the cells were incubated for 10 min with propidium iodide (PI) (Sigma-Aldrich, Dorset, Germany) at a final concentration of 20 μg.mL^-1^ to assess the loss of membrane integrity.

### Confocal imaging

Yeast cells were imaged with an Olympus FluoView 1000 inverted confocal laser scanning microscope, using a 100x UPlanApo objective lens, NA 1.40. AMAC fluorescence was imaged using excitation at 405 nm and with emission bandpass filters set to 490–560 nm. PI fluorescence was imaged using excitation at 559 nm and a 570 nm long pass emission filter. Transmission light images used excitation at 473 nm. Images were acquired using a linescanning Kalman filter of 5 within the FluoVew 1000 software to improve the signal-to-noise ratio, and processed using ImageJ [25].

### Statistical analysis

Experiments were done at least in duplicate. Experimental data was analyzed using Minitab version 14.1 (Minitab 14, State College, PA). Student’s *t-*tests were performed to identify differences between samples. Differences were considered to be significant when *p* ≤ 0.05.

## Results

**Fig 1:**
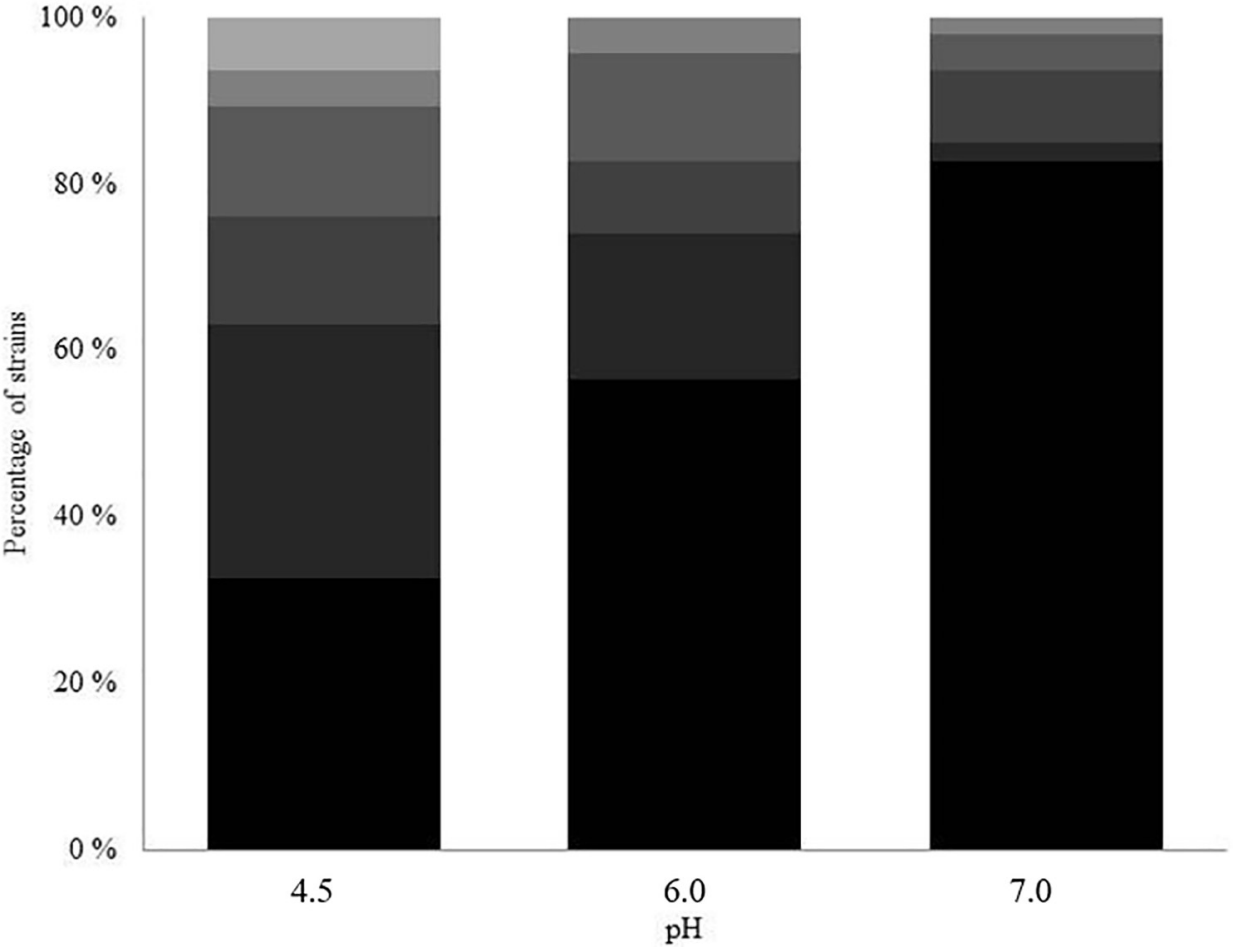
Distribution of MIC values for C32 among 52 tested yeast strains. The graph shows the percentages of strains having MIC values >5000 μg mL^-1^ (dark), 5000–1250 μg mL^-1^, 625–156 μg mL^-1^, 78–39 μg mL^-1^, 19.5–9.8 μg mL^-1^ and <9.8 μg mL^-1^ (light) at different pH, with n = 52.

The antifungal activity of CHOS with polymerization degree (DP_n_) of 32 and fraction of acetylation (F_A_) of 0.15 (C32) was studied on 52 medically relevant yeast strains. Fig 1 shows an overview of the minimal inhibitory concentrations measured for the 52 tested strains, at pH 4.5, 6.0 and 7.0. The antifungal effect of C32 was maximal at pH 4.5 and showed a clear reduction at elevated pH. The MIC values varied widely among the yeast strains (from > 5000 μg.mL^-1^ to < 9.77 μg.mL^-1^). Clinical isolates showed higher MIC values compared to ATCC strains (results not shown). *R*. *mucilaginosa* was especially sensitive, having a MIC < 9.77 μg mL^-1^ at pH 4.5. Conversely, some strains, including *C*. *albicans* and *S*. *pombe*, were resistant (MIC > 5000 μg mL^-1^) at all pH values.

Table 1 shows the distribution of MFC/MIC ratios, i.e. the ratio between the minimal fungicidal concentration and the minimal inhibitory concentration among the 52 yeast isolates. The MFC/MIC values were generally lower than 4, suggesting that C32 has fungicidal rather than fungistatic activity [24].

**Table 1:** MFC/MIC ratios for inhibition of clinically relevant yeast strains by C32 at pH 4.5, 6 and 7. For an overview of MIC values, see Fig 1. Note that this ratio could not be calculated for strains with a MIC < 9.77 (“Low MIC”), and for strains with a MIC close to or larger than 5000 μg mL^-1^ (“High MIC”).

	Percentage of strains with MFC/MIC ratio					
pH	1	2	4	8	Low MIC	High MIC
4.5	49	8			6	37
6.0	29	10				61
7.0	8	6		2		84

**Fig 2:**
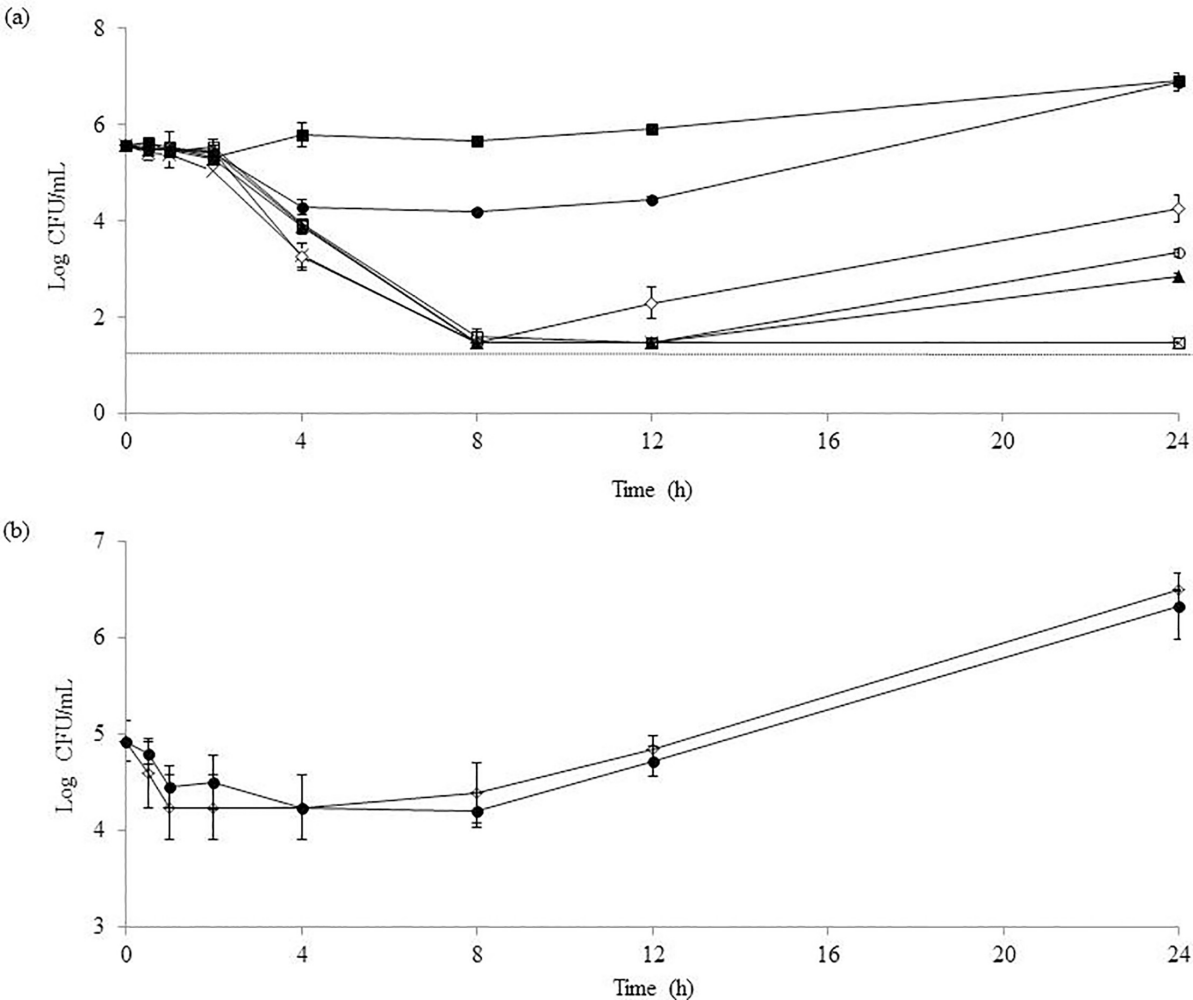
Inhibition of *Candida spp*. over time. The yeast (<number> CFU) was incubatd for 24 hours in <medium/buffer>, pH 4.5, containing C32, at <temperature> and samples were taken to determine the number of viable cells. (a) *C*. *guilliermondii* counts (average ± standard deviation) for control (■), 0.25 x MIC (●), 0.5 x MIC (◊), 1 x MIC (○), 2 x MIC (▲), 5 x MIC (x), 10 x MIC (□). The dotted line represents the detection limit of the method for detecting viable cells. (b) *C*. *albicans* counts (average ± standard deviation) for an incubation with of 5000 μg mL^-1^ of C32 (●) and a control incubation (◊).

In an effort to further understand the relationship between the concentration of C32 and its antifungal activity, time-kill studies were conducted on the C32-susceptible clinical isolate *C*. *guilliermondii* 12146, using doses above and below the MIC (Fig 2A). C32 showed time and dose-dependent inhibitory effects. The curves show that there is a delay of around 2 h after the addition of the CHOS, before killing of the cells becomes detectable. At all C32 concentrations, the number of surviving cells was drastically reduced within the first 8 hours, followed by a slow and clearly dose-dependent recovery. For the highest C32 concentrations tested (10 and 5 x MIC), no growth was detected even after 24 h of incubation. Notably, for all C32 concentrations ≥ 0.5 x MIC cell counts where significantly lower (*p* ≤ 0.05) than in the control after 24 h of incubation. Similarly, the effect of C32 (5000 μg mL^-1^) on the growth of the C32-resistant clinical isolate *C*. *albicans* 1581 was studied along 24h (Fig 2B). C32 did not significantly affect the viability of the strain, not even during the first 8 h of incubation.

**Fig 3:**
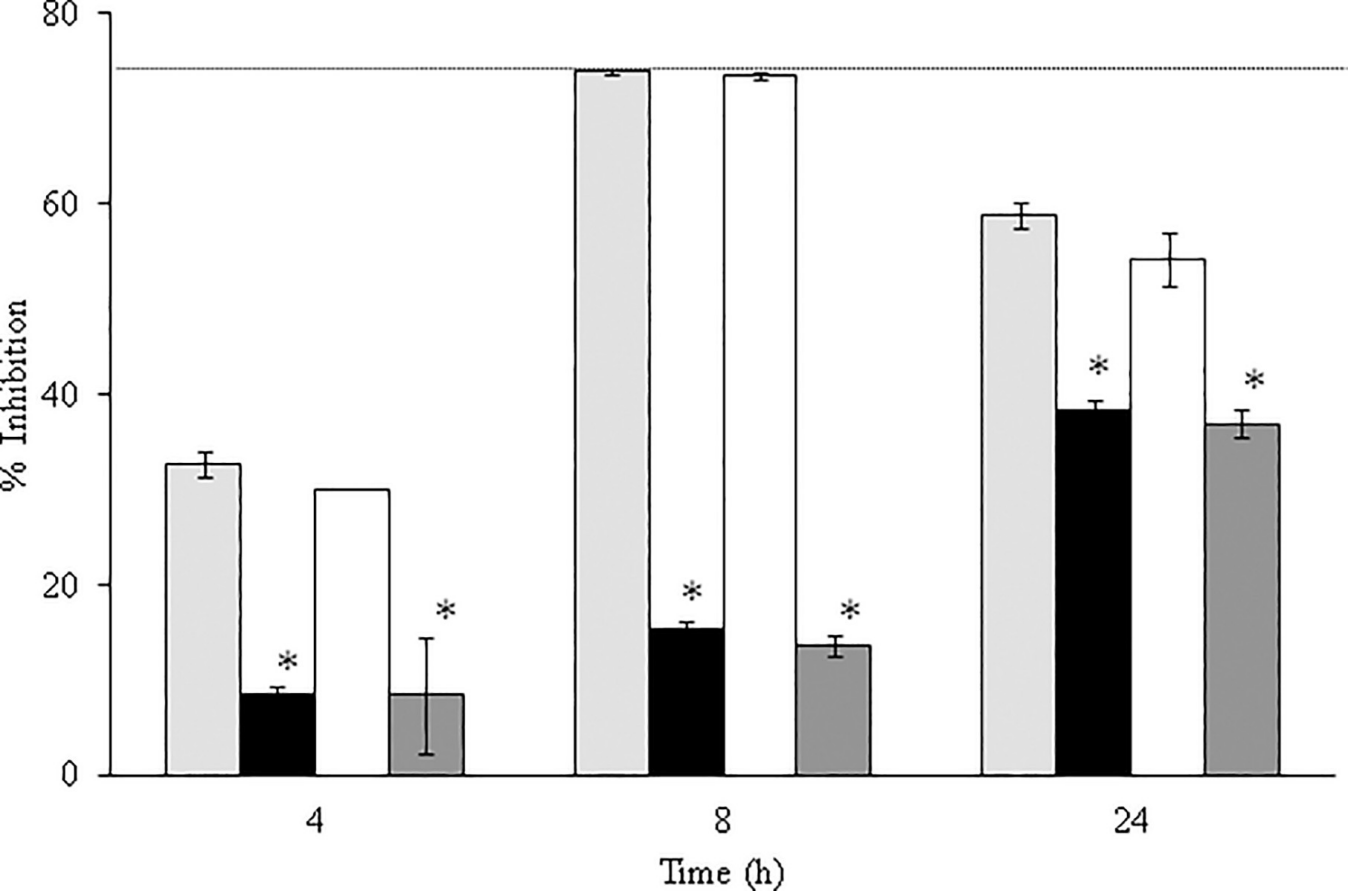
Inhibition of *C*. *guilliermondii* in the presence of C32, at 1 x MIC, at pH 4.5 (average ± standard deviation). Both non pretreated cells and celles recovered from a C32 culture were tested: light grey bars, not pretreated, 37 ^o^C; black bars, not pretreated, 41.5 ^o^C; white bars, recovered, 37 ^o^C; dark grey bars, recovered, 41.5 ^o^C. *, Significantly different from “not pretreated, 37 ^o^C” (*p* < 0.05).

The *C*. *guillermondii* 12146 colonies recovered after 24 h of exposure to C32 were smaller and had a slower growth rate than colonies obtained from non-exposed cells. Such colonies were used to inoculate fresh medium and growth was compared to growth in cultures inoculated with non-treated cells, at physiological temperature (37°C) and fever temperature (41.5°C) (Table 2). Cells grown at fever temperatures showed smaller growth rates. However, these tests did not reveal significant differences (*p* > 0.05) between the growth potential of treated and non-treated cells. Cultures inoculated with treated or non-treated cells were then exposed to C32 at 1 x MIC at both temperatures, revealing no significant differences in C32 sensitivity (*p* > 0.05; Fig 3).

**Table 2:** Growth of *C*. *guilliermondii* with or without pretreatment with C32 at standard (37°C) and fever temperature (41.5°C). Growth after 4 h, 8 h and 24 h is expressed relative to the growth of non-pretreated cells at 37°C. See text for further details.

	% of growth		
Time (h)	Not pretreated, 41.5°C	Pretreated, 37°C	Pretreated, 41.5°C
4	92 ± 4^a^	96 ± 3	93 ± 8^a^
8	87 ± 1^a^	98.6 ± 0.1	87.3 ± 0.3^a^
24	88 ± 2^a^	96 ± 4	87 ± 2^a^

^a^ Significantly different to PSPT (*p* < 0.05)

**Fig 4:**
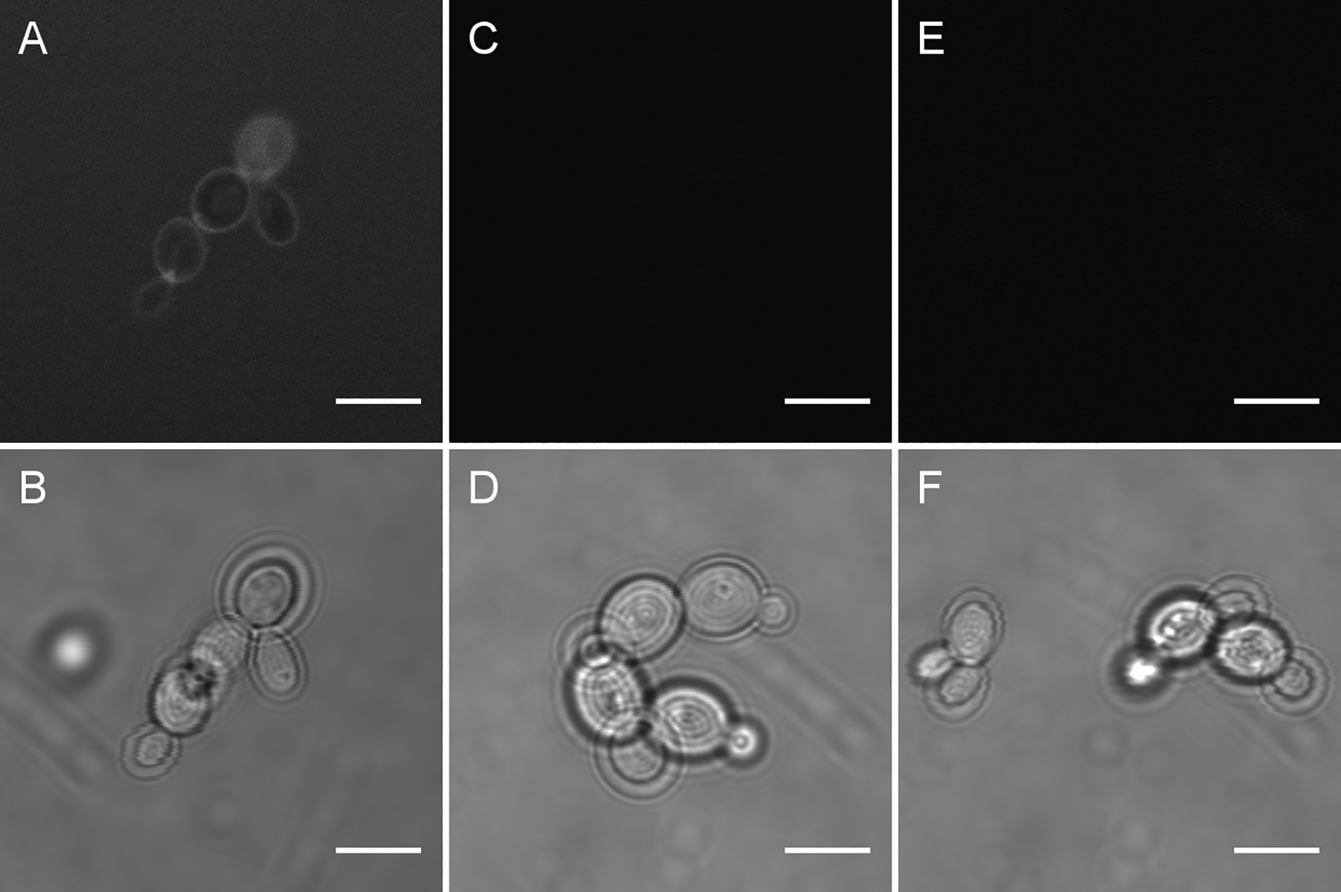
AMAC fluorescence (upper panel A, C, E) and transmission image (lower panel B, D, F) for *C*. *guillermondii* cells incubated for 24 hours with AMAC-C32 (A, B), unlabeled C32 (C,D) or without C32 (E,F). The concentration of C32 was equal to the minimal inhibitory concentration. The scale bar represents 5μm.

**Fig 5:**
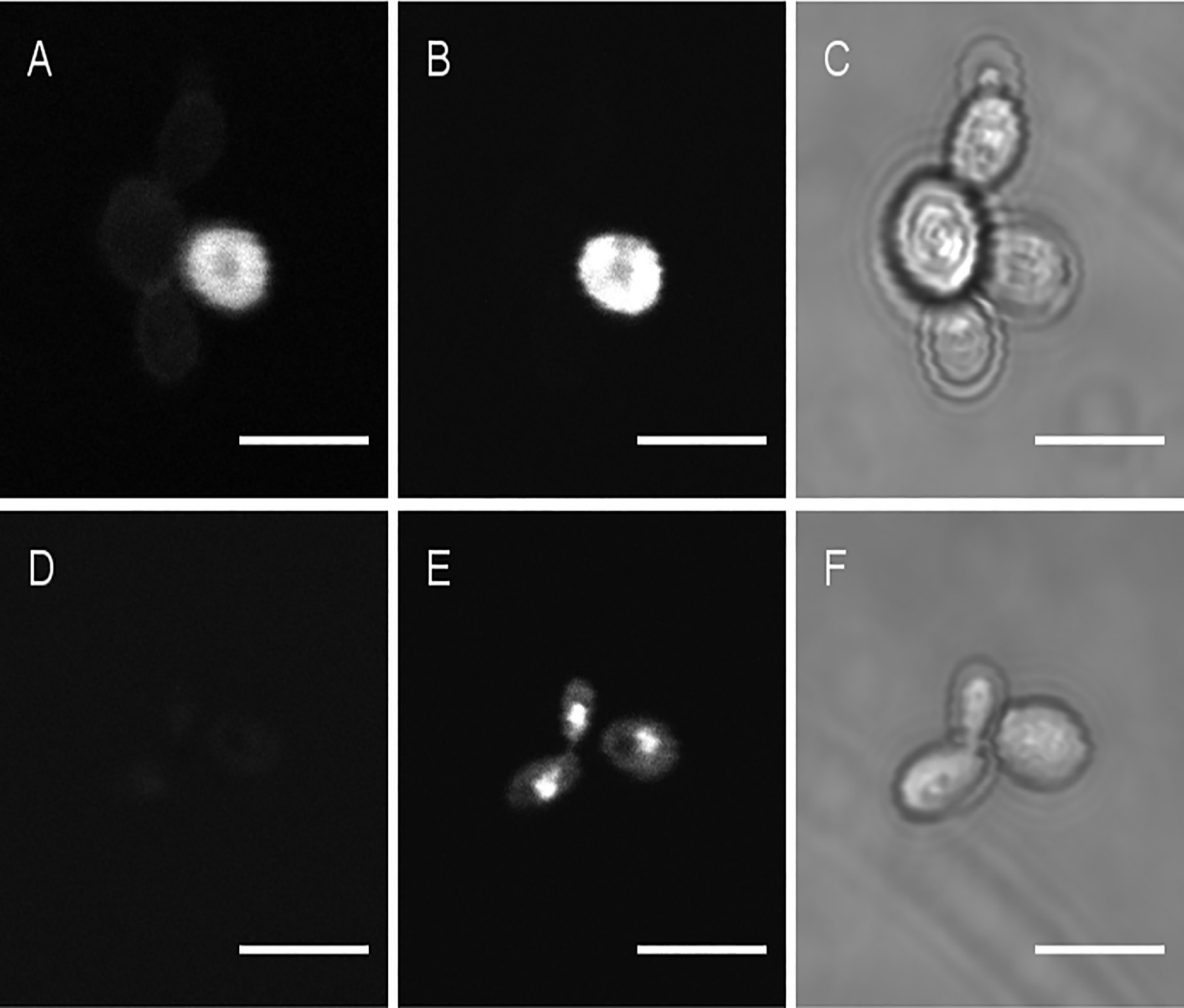
AMAC fluorescence (left panels, A and D), propidium iodide (PI) fluorescence (central panels, B and E) and transmission image (right panels, C and F) for *C*. *guillermondii* cells incubated for 24 hours with AMAC-labeled C32 (A, B, C) in RPMI medium. Ten minutes treatment with 50% ethanol (D, E, F) to provide a positive control for PI staining. The concentration of C32 was equal to the minimal inhibitory concentration. Scale bars represent 5μm.

Confocal imaging of *C*. *guillermondii* exposed to AMAC-labeled C32 showed that C32 adsorbs to the cell surface. Interestingly, when using C32 at concentrations ≥ MIC we consistently observed two different fluorescent patterns; some cells seemed to have their surfaces covered by C32, whereas other cells gave the impression of C32 being present inside the cells (Fig 4A). Propidium iodide (PI), a membrane impermeant dye is generally excluded from viable cells but that can penetrate cell membranes of dying or dead cells, was then used to analyze cell membrane integrity (Fig 5). Confocal fluorescence images show a much narrower focal section than transmission images, so the fluorescence is only in focus for parts of each transmission image. Cells showing seemingly intracellular AMAC-C32 showed the strongest labeling with PI-labeled cells (exemplified in Fig 5A and 5B; similar additional results not shown), suggesting that the cells giving the highest AMAC-C32 signals have disrupted cell membranes and are possibly dead. Fig 5E shows that unlabeled C32-treated cells also display increased permeability to PI, indicating membrane disruption. Similar PI staining was observed in positive control cells (Fig 5H) in which cell membranes were previously disrupted with 50% ethanol. Taken together, the data presented in Fig 5 show that C32 adsorbs to the cell surface, disturbs membrane integrity, and may accumulate inside the cells.

**Fig 6:**
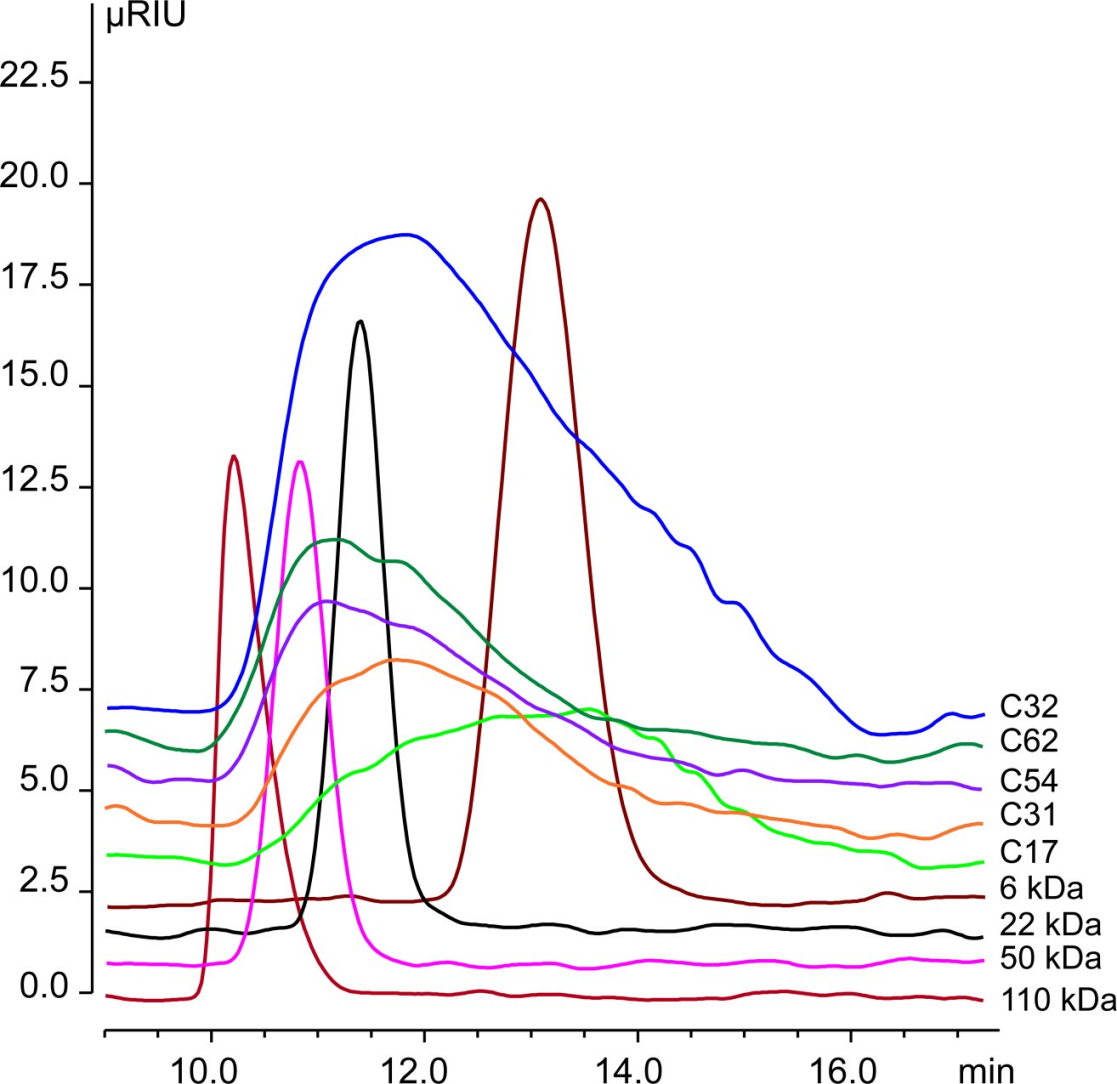
Size-exclusion chromatograms of CHOS preparations and pullulan standards.

To assess the size-dependency of the inhibitory effects of CHOS on fungal growth, the C32 preparation was fractionated by size-dependent dialysis. Several new mixtures were prepared: i) below 3.5 kDa, ii) between 3.5 kDa and 8.0 kDa, iii) above 3.5 kDa, and iv) above 10 kDa. The average degree of polymerization (DP_n_) was determined for each fraction by ^1^H NMR [20] (Table 3). Moreover, analytic size-exclusion chromatography was used to determine the relative molecular weight average (MW) (Table 3 and Fig 6).

**Table 3:** Properties of CHOS fractions derived from ^1^H-NMR and SEC.

Fraction	C32	C17	C31	C54	C62
cutoff (kDa)^*a*^	n.a.	< 3.5	>3.5 <8	>3.5	>10
^1^H-NMR (DP_n_)^*b*^	32	17	31	54	62
MW (kDa)^*c*^	15	7.6	15	21	24

^*a*^ Dialysis cut-offs used to separate C32 into different CHOS preparations.

^*b*^ Standard NMR method used to characterize CHOS fractions. These DP_n_ values are used to name the CHOS fractions.

^*c*^ Weighted average molar mass are calculated relative to pullulan standards assuming linearity in the entire calibration range (from 6 kDa to 110 kDa) and below.

Table 4 shows the antifungal effects of the various CHOS fractions. In line with the results described above, *C*. *albicans* was resistant to all the fractions. For the rest of the strains tested, the trend was that fractions with an intermediate degree of polymerization, i.e. C31, and C54, had the strongest inhibitory effects. Of the well-characterized samples (Table 3), C31 and C54 were clearly more effective than C17 and C61 (Table 4).

**Table 4:** Effect of CHOS with different DP_n_ on the growth of medically relevant yeasts. Results are expressed as minimum inhibitory concentration (MIC, μg mL^-1^).

		Strain			
Cutoff^a^	DP_n_^b^	*C*. *albicans*	*C*. *guillermondii*	*C*. *lusitaneae*	*C*. *parasilopsis*
< 3.5	17	>5000	78	156	19.5
> 3.5 < 8	31	>5000	39	78	4.9
> 3.5	54	>5000	39	78	4.9
>10	62	>5000	1250	2500	1250

^a^ kDa

^b^ as determined by ^1^H NMR; the four fractions with a DP_n_ value are named by this value and further details on their characterization appear in Table 3.

## Discussion

The data presented above show that the antifungal effect of C32 against *Candida* spp varies greatly among strains and isolates, with clinical isolates generally showing lower sensitivity. It is conceivable that modifications in the cell membrane of clinical isolates increase their resistance to the CHOS [26]. Reversible adaptive resistance is a commonly reported but little understood phenomenon, which entails that an antifungal becomes less effective as a fungus is repetitively exposed. This effect has been reported to have finite duration and both the duration and the strength (i.e. the level of resistance) seem to be dependent on the antifungal concentration at the time of the preceding exposure [27, 28]. While this phenomenon has been often described for other antifungals, our data show that this phenomenon is absent in the C32-susceptible strain *C*. *guillermondii*.

The observed pH-dependency of the antifungal effect of C32 is not surprising since similar results have been reported for chitosan [12, 16]. The pH-dependency is likely primarily due to the fact that the molecule becomes more polycationic at lower pH (the p*K*_a_ of the amino group in glucosamine is 6.3–6.5 [29]). The more cationic the CHOS, the stronger they may interact with negatively charged microbial components (membrane components, DNA, RNA). It has been recently reported that some of the most used antifungals (amphotericin B, azoles and triazoles) are not as effective in acidic condition as at physiological pH [30]. Thus, CHOS may be a particularly attractive alternative for known anti-fungals for the treatment of yeast infections in low pH compartments of the body.

The present results further show that the environmental temperature affects the yeast’s susceptibility to CHOS. It has been reported that thermal stress can change the membrane composition and fluidity of *C*. *albicans*, increasing its chitin content and altering the expression of remedial wall remodeling enzymes [31]. It is possible that these known temperature effects on the yeast cells relate to the observation that the cells are less susceptible to C32 at 41.5°C compared to 37°C.

Effective antifungal therapy is currently very limited and is mainly dominated by therapies based on administering azoles [32, 33]. Members of this class of antifungals are fungistatic rather than fungicidal. When fungi are exposed to fungistatic agents, cells enter a non-growth-survival phase in which viability is not affected but new growth is inhibited [34]. In general, fungistatic substances limit the growth by interfering with protein production, DNA replication, and other aspects of cellular metabolism. In contrast, fungicidals lead to cell death, thus possibly providing a more permanent effect. Fungicidal drugs are scarce. Still, they are often preferred over fungistatic ones especially for topical therapies [35, 36].

Even though the antimicrobial activity of chitosan has been widely studied, its exact mechanism of action is not fully understood [12] and there are contradictory opinions regarding its static or killing effect [37–39]. The dose-response curves depicted in Fig 2 may be taken to suggest that C32 has both fungistatic and fungicidal effects, at lower and higher C32 concentrations, respectively, but the more likely explanation for the curves is that C32 efficacy is reduced over time due to it being metabolized and that this takes longer time at higher C32 dosages. Indeed, other data showed that the effect of C32 generally is fungicidal (Table 1) and that cells growing in recovering C32-treated cultures had traits similar to cells that had never seen C32, including similar sensitivity for C32 (Fig 3). It is interesting to note the two-hour delay in the inhibitory effect of C32 on *C*. *guilliermondii* (Fig 2). This delay suggest that the time for other antifungals to act can increase, and needs to be taken into account while designing anti-fungal therapies involving C32. Although, under the conditions used here, C32 largely acted in a fungicidal manner, it is likely that fungistatic effects occur, especially at lower concentrations. In a recent work, Peña *et al*. [15] studied the concentration dependency effect of chitosan (96 kDa) against a strain of *Candida albicans* (ATCC 10231). At low concentrations chitosan binds to the fungal cell membrane, producing K^+^ efflux, extracellular acidification, an increased transmembrane potential and increased uptake of Ca^2+^. The authors further proposed that these mild effects, translating in inhibition of growth, are due to a decline of the negative charge on the cell surface resulting from the binding of chitosan. The study further showed that higher concentrations of chitosan lead to large efflux of phosphates and decrease in Ca^2+^ uptake in addition to growth inhibition [15].

Interestingly, the data presented above show that the antifungal effect of CHOS depends on DP_n_, and that fractions with a DP_n_ in the 30–50 range are most active. In a previous study, Rahman *et al*. [40] studied the effect of a series CHOS with largely differing DP_n_ (DP_n_ = 3–206) on germination of *Botrytris cinerea* and *Mucor piriformis*. The authors found that CHOS with DP_n_ 23 and 40 had the highest inhibitory effect on germination of the tested pathogens.

The possibility of producing well-defined CHOS such as C32 opens new perspectives for these compounds to be used as antifungals in medical therapy. Their low toxicity and high activity supports their use in different applications, especially in environments with low pH, such as the vagina. Since the *in vitro* efficacy of drugs not necessarily reflects their effects *in vivo*, further *in vivo* studies on the antimicrobial activity of chitosan alone and in combination with other antifungals are needed in order to investigate their use as an alternative to current therapies for treating yeast infections in humans.

## Conclusions

This study demonstrates the potential of a well-defined chitosan oligosaccharide mixture (CHOS, C32) to be used for treating yeast infections in humans. The results stress the influence of the physiological state of the patient (body temperature) and infection location [41] on the antifungal effect of C32. The study also outlines the differences on the resistance patterns among yeast species, strains and isolates. The results suggest that the antifungal activity of C32 is mainly fungicidal, caused by the adsorption of C32 to the cell surface, with the consequent promotion of the cell disruption and accumulation in the cytoplasm. Further studies are needed in order to define the feasibility of C32 to be used as a therapy for human fungal infections.
